# The loss of dopaminergic neurons in DEC1 deficient mice potentially involves the decrease of PI3K/Akt/GSK3β signaling

**DOI:** 10.18632/aging.102599

**Published:** 2019-12-28

**Authors:** Zhu Zhu, Wu Yichen, Zhang Ziheng, Ge Dinghao, Lu Ming, Liu Wei, Shan Enfang, Hu Gang, Hiroaki Honda, Yang Jian

**Affiliations:** 1 Department of Pharmacology, Nanjing Medical University, Nanjing, China; 2 Department of Pharmacology Sciences, Nanjing University of Chinese Medicine, Nanjing, China; 3 Research Institute for Radiation Biology and Medicine, Hiroshima University, Hiroshima, Japan

**Keywords:** differentiated embryonic chondrocyte gene 1 (DEC1), dopaminergic (DA) neurons, Parkinson's disease (PD), PI3K/Akt/GSK3β signaling

## Abstract

Here we study the effects of differentiated embryonic chondrocyte gene 1(DEC1) deficiency on midbrain dopaminergic(DA) neurons in the substantia nigra pars compacta(SNpc) through behavioral, histological and molecular analysis. We have found that compared to the age-matched WT mice, DEC1 deficient mice show a decrease in locomotor activity and motor coordination, which shows the main features of Parkinson’s disease(PD). But there is no significant difference in spatial learning and memory skills between WT and DEC1 KO mice. Compared to the age-matched WT mice, DEC1 deficient mice exhibit the loss of DA neurons in the SNpc and reduction of dopamine and its metabolites in the striatum. The activated caspase-3 and TH/TUNEL^+^ cells increase in the SNpc of 6- and 12-month-old DEC1 KO mice compared to those of the age-matched WT mice. But we haven't found any NeuN/TUNEL^+^ cell increase in the hippocampus of the above two types of mice at the age of 6 months. Furthermore, DEC1 deficiency leads to a significant inhibition of PI3K/Akt/GSK3β signaling pathway. Additionally, LiCl could rescue the DA neuron loss of midbrain in the 6-month-old DEC1 KO mice. Taken together, the loss of DA neurons in the DEC1 deficient mice potentially involves the downregulation of PI3K/Akt/GSK3β signaling.

## INTRODUCTION

Parkinson’s disease (PD), a common, age-related neurodegenerative disorder, is characterized by motor symptoms, including bradykinesia, resting tremor, rigidity, gait disturbance and postural instability. These motor defects are due to a progressively preferential loss of midbrain dopaminergic (DA) neurons in the substantia nigra pars compacta (SNpc), which subsequently results in the decreased level of dopamine in the striatum [1–3]. Strong oxidative stress, reduced antioxidant levels and mitochondrial defects eventually induce neuronal death in cellular systems involving the pathogenesis of PD [4]. Aging is acknowledged to be a primary risky factor to the development of PD. And PD is considered to be able to affect more than 2% of the population after 65-year-old [5]. Many studies have revealed the crucial roles of several transcription factors and nuclear cofactors in the midbrain DA neuron development. The transcriptional factors such as the orthodenticle homeobox 2(Otx2), pre-B-cell leukemia homeobox 1(PBX1), and LIM domain-binding protein 1(Ldb1) are essential to the development of the midbrain DA neurons, but they may be impaired in PD [6–8]. Understanding how the cellular mechanisms mediate the loss of DA and how these mechanisms affect DA neurons may provide insight into the prevention and treatment of PD.

Human differentiated embryonic chondrocyte gene 1 (DEC1) (also known as Stra13 and Sharp2), a basic helix-loop-helix (bHLH) transcription factor, is involved in various cellular events such as cell proliferation and differentiation, circadian rhythm and lipid metabolism [9–12]. It is expressed widely in normal tissues and its expression is rapidly induced in response to different stimuli such as growth factor, light pulse or hypoxia [9, 11, 13]. Studies have demonstrated that aged DEC1^-/-^ mice developed autoimmune disease, a gross enlargement of spleen, thymus, and lymph nodes [14, 15]. DEC1 is also reported to have anti-apoptotic effect by decreasing caspases and increasing survivin [16, 17]. Furthermore, overexpression of DEC1 promoted neuronal differentiation of P19 cells and DEC1 expression could be rapidly activated by nerve growth factor (NGF) in PC12 cells [9, 18]. In the previous study, we have reported that there is much expression of DEC1 in the midbrain. And the expression of DEC1 and tyrosine hydroxylase (TH) decreases in the SNpc after the administration of 1-methyl-4-phenyl-1, 2, 3, 6-tetrahydropyridine (MPTP) in mice [19]. In addition, we have demonstrated that the downregulation of DEC1 contributes to MPP^+^-induced apoptosis in SH-SY5Y cells [19]. However, there is little knowledge about the physiological role of DEC1 in DA neuron degeneration in age-related PD.

In this study, we used different age (3-, 6- and 12-month-old) groups of DEC1 knockout (KO) mice to investigate the influence of DEC1 deficiency on the behavioral tests, the number of DA neurons in the SNpc, TH and dopamine transporter (DAT) expression and the apoptosis-related markers in the midbrain. We further determined the expression of β-catenin, one of the critical transcriptional factors in DA neuron developmental process [20], and the involvement of phosphatidylinositol 3-kinase p110α (PI3Kp110α), protein kinase B (PKB or Akt) and glycogen synthase kinase 3 β(GSK3β).

## RESULTS

### DEC1 deficiency shows motor abnormalities in mice

**Figure 1:**
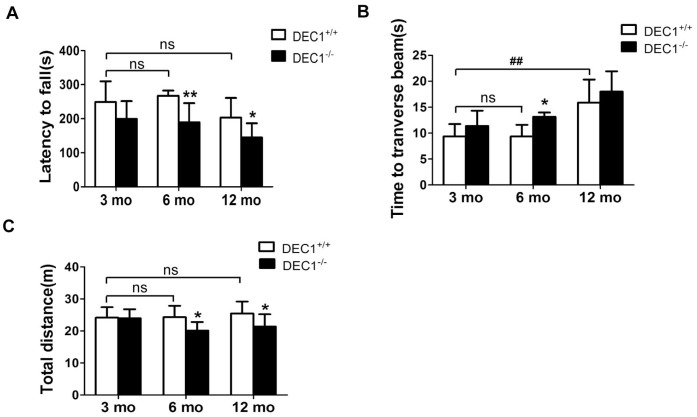
**DEC1 deficient mice exhibit motor abnormalities.** (**A**–**C**) Locomotion activity and motor coordination were analyzed by rotarod test (RT), beam walking test (BWT) and open-field test (OFT) in DEC1^+/+^ and DEC1^-/-^ mice (n=15 in each group) at the age of 3, 6 and 12 months. (**A**) The latency to fall off the rotated rod in RT (Two-way AONVA, gene: F_(1,54)_=17.767, p<0.001; age: F_(2,54)_=6.041, p=0.004; interaction: F_(2,54)_=0.297, p= 0.744). (**B**) Time to transverse the beam within 5 min in BWT (Two-way AONVA, gene: F_(1,40)_=7.354, p=0.001; age: F_(2,40)_=20.977, p<0.001; interaction: F_(2,40)_=0.288, p= 0.751). (**C**) Total traveled distance (Two-way AONVA, gene: F_(1,49)_=9.333, p=0.004; age: F_(2,49)_=1.266, p=0.291; interaction: F_(2,49)_=2.274, p= 0.114). The data are analyzed using t-test for the same age in two genotypes of mice and expressed as mean ± SD. *p<0.05, **p<0.01 vs the age-matched littermate DEC1^+/+^ mice. ## p<0.01, ns p>0.05, comparisons are shown in the figure.

The locomotion ability and motor coordination were explored at the age of 3, 6 and 12 months in DEC1^+/+^ and DEC1^-/-^ littermate mice. Rotarod test (RT) is considered to be a reliable test to estimate motor coordination ability. As shown in Figure 1, compared with that in the age-matched DEC1^+/+^ mice, the latency on the rotated rod in 6- and 12-month-old DEC1^-/-^ mice was markedly reduced by 29.1% and 28.8%, respectively (p<0.01, 0.05) (Figure 1A). But the latency on the rotated rod in 3-month-old DEC1^-/-^ mice did not decrease significantly compared to that in age-matched DEC1^+/+^ littermate mice (p>0.05) (Figure 1A). In the beam walking test (BWT), the prolongation of the walking time to traverse the beam in the 6-month-old DEC1^-/-^ mice increased significantly by 40.5% compared to that in the age-matched DEC1^+/+^ mice (p<0.05) (Figure 1B). We noted that the walking time to traverse the beam in 12-month-old WT mice increased significantly compared with that in 3-month-old WT mice(p<0.01) (Figure 1B). Spontaneous activity was examined by the open-field test (OFT). As shown in Figure 1C, the traveled distance in 6- and 12-month-old DEC1^-/-^ mice decreased by 17.3% and 16.1% respectively, compared to that in age-matched DEC1^+/+^ littermate mice (p<0.05), but the traveled distance in 3-month-old DEC1^-/-^ mice did not decrease significantly compared to that in age-matched DEC1^+/+^ littermate mice (p>0.05).

In order to investigate whether DEC1 deficiency could cause spatial learning and memory deficits, morris water maze task (MWM) was conducted in DEC1^+/+^ and DEC1^-/-^ littermate mice at the age of 3, 6 and 12 months. As shown in Supplementary Figure 1, although both two types (WT, KO) of mice at the age of 12 months showed greater decreased spatial learning and memory skills than those at the age of 3 and 6 months do, the escape latency required to find the hidden platform had no significant difference for those at the age of 3, 6 and 12 months between the two genotypes of mice (p>0.05) (Supplementary Figure 1A). Subsequently, a probe test was performed by removing the platform to measure the strength of the memory trace. As a result, the swimming time in the target quadrant and the number of mice passing over the platform area showed no significant difference for those at the age of 3, 6 and 12 months between the two genotypes of mice (p>0.05) (Supplementary Figure 1B, 1C). The data implied that DEC1 deficiency failed to impact spatial learning and memory in 3~12-month-old mice. Taken together, these data demonstrated that DEC1 deficiency was able to trigger defects in the locomotor activity and motor coordination, but not in the spatial learning and memory in mice.

### DEC1 deficient mice show the loss of DA neurons in the SNpc

**Figure 2:**
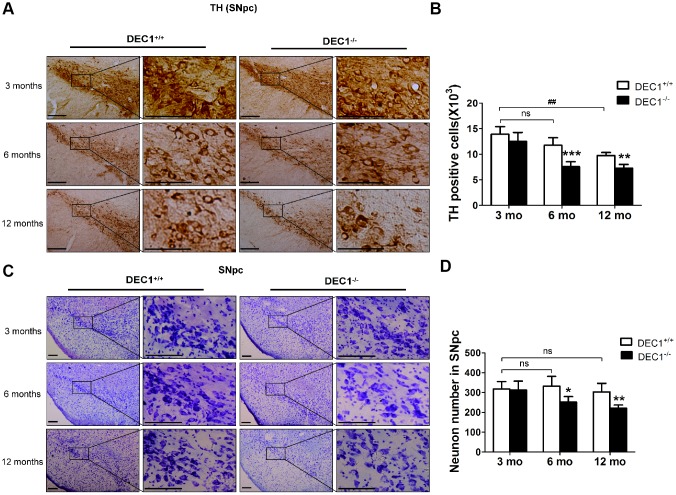
**DEC1 deficient mice show the loss of DA neurons in the SNpc.** (**A**) Immunohistochemical staining of TH^+^ DA neurons in the SNpc of DEC1^+/+^ and DEC1^-/-^ mice at the age of 3, 6, 12 months (n=6 in each group). (**B**) Stereological counts of TH^+^ cells in the SNpc of DEC1^+/+^ and DEC1^-/-^ mice at the age of 3, 6 and 12 months (Two-way AONVA, gene: F_(1,28)_=37.088, p<0.001; age: F_(2,28)_=34.758, p<0.001; interaction: F_(2,28)_=3.789, p= 0.035). (**C**) Nissl staining in the SNpc in DEC1^+/+^ and DEC1^-/-^ mice at different ages of 3, 6 and 12 months (n=6 in each group). (**D**) Quantification of Nissl staining in the SNpc of DEC1^+/+^ and DEC1^-/-^ mice at 3, 6 and 12 months(n=6 in each group) (Two-way AONVA, gene: F_(1,22)_=14.78, p=0.01; age: F_(2,22)_=4.515, p=0.023; interaction: F_(2,22)_=3.132, p=0.064). Neurons were imaged and counted with an Olympus DP70 microscope (×100 or ×200). The data are analyzed using t-test for the same age in two genotypes of mice and expressed as mean ± SD. *p<0.05, **p<0.01, ***p<0.001 vs the age-matched DEC1^+/+^ mice. ## p<0.01, ns p>0.05, comparisons are shown in the figure. Scale bar=100 μm.

Since DEC1 is highly expressed in sub-regions of brain and is an important regulator of apoptosis and proliferation [16–18], we determined whether the reduced locomotor ability and motor coordination of DEC1^-/-^ mice were correlated with the alterations in the histology of DA neurons in the SNpc. We detected the number of TH positive cells in the two types (DEC1^+/+^ and DEC1^-/-^) of mice at the age of 3, 6 and 12 months using immunohistochemical staining with an antibody against TH. As shown in Figure 2, the number of TH^+^ neurons in the SNpc significantly decreased by 35.6% and 25.3% in the 6- and 12- month-old DEC1^-/-^ mice, compared with that in the age-matched littermate DEC1^+/+^ mice (p<0.001, 0.01) (Figure 2A, 2B). Whereas the number of TH^+^ neurons in the SNpc did not significantly decrease in the 3-month-old DEC1^-/-^ mice, compared with that in the age-matched littermate DEC1^+/+^ mice (p>0.05) (Figure 2A, 2B). To determine whether DEC1 deficiency caused neurodegeneration is specific in the SNpc, we determined survival neurons in different brain regions such as the midbrain and hippocampus, with Nissl staining. The number of neurons in the SNpc in the 6- and 12-month-old DEC1^-/-^ mice decreased by 24.3% and 27.1% compared with that in the age-matched DEC1^+/+^ mice (p<0.05, 0.01) (Figure 2C, 2D), whereas the neuron number of the SNpc was not significant between the two types of mice at the age of 3 months (p>0.05) (Figure 2C, 2D). But, the number of neurons in the hippocampus showed no significant difference between the two types (DEC1^-/-^ mice and DEC1^+/+^) of littermate mice at the age of 3, 6 and 12 months (Supplementary Figure 3A, 3B). The results supported that DEC1 deficiency might cause neuronal loss in a cell type specific manner.

**Figure 3:**
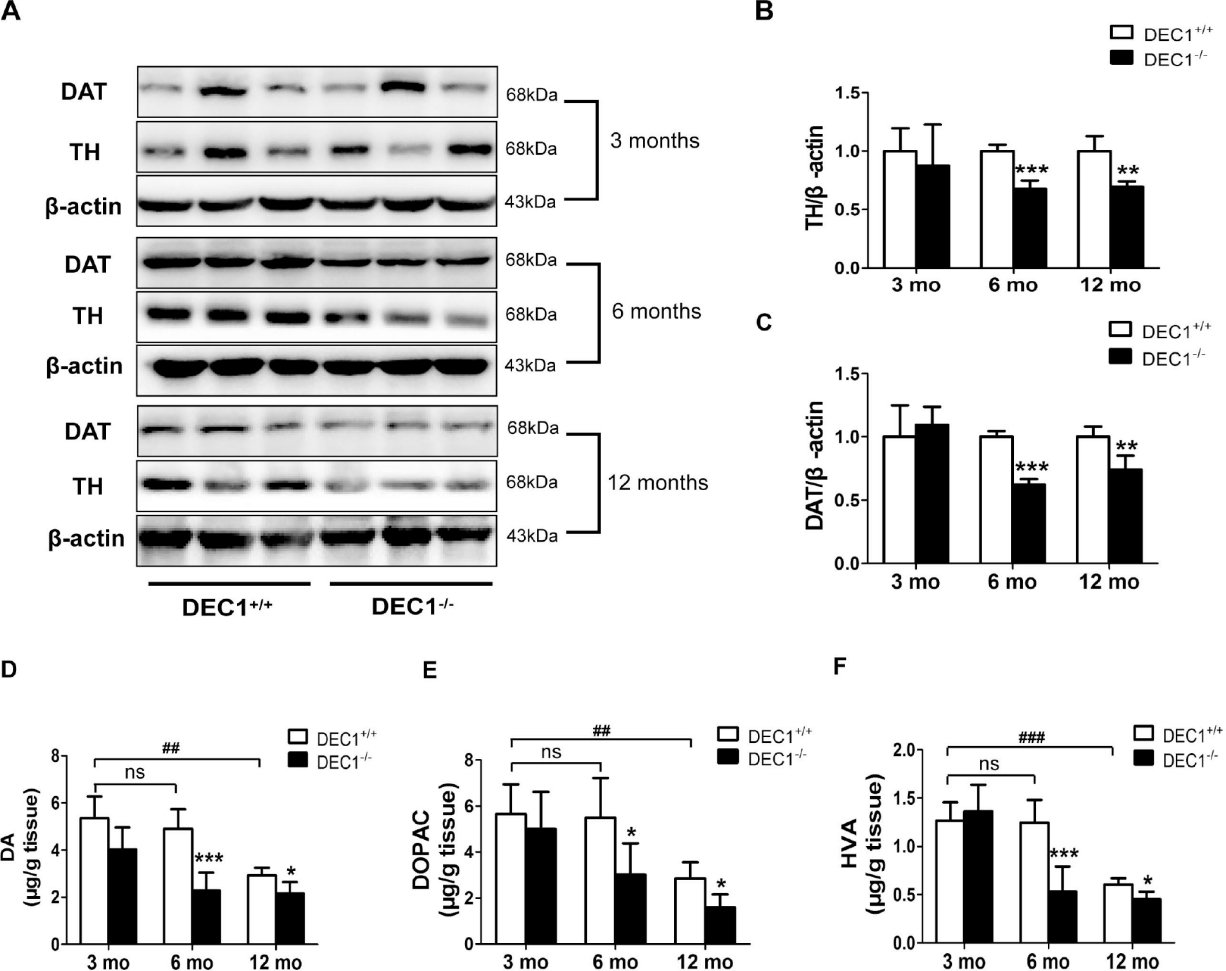
**DEC1 deficient mice exhibit a decrease of TH and DA neurons in the midbrain**. (**A**) TH and DAT expression in the midbrain of DEC1^+/+^ and DEC1^-/-^ mice (n=6 in each group) at the age of 3, 6, 12 months using Western blotting. (**B**) TH/β-actin (Two-way AONVA, gene: F_(1,16)_=8.618, p=0.001; age: F_(2,16)_=0.963, p=0.403 interaction: F_(2,16)_=3.019, p= 0.077). (**C**) DAT/β-actin (Two-way AONVA, gene: F_(1,18)_=9.519, p=0.002; age: F_(2,18)_=12.897, p=0.02; interaction: F_(2,18)_=2.832, p= 0.185). (**D**–**F**) The amount of dopamine (DA), dihydroxyphenylacetic acid (DOPAC), and homovanillic acid (HVA) in the striatum of DEC1^+/+^ and DEC1^-/-^ mice at the age of 3, 6, 12 months using HPLC (n=6 in each group). (**D**) DA (Two-way AONVA, gene: F_(1,25)_=32.562, p<0.001; age: F_(2,25)_=16.683, p<0.001; interaction: F_(2,25)_=4.247, p= 0.272). (**E**) DOPAC (Two-way AONVA, gene: F_(1,25)_=9.216, p=0.006; age: F_(2,25)_=0.963, p<0.001; interaction: F_(2,25)_=1.373, p= 0.026). (**F**) HVA (Two-way AONVA, gene: F_(1,25)_=11.539, p=0.006; age: F_(2,25)_=33.317, p<0.001; interaction: F_(2,25)_=10.872, p<0.001). The data are analyzed using t-test for the same age in two genotypes of mice and expressed as mean ± SD. *p<0.05, **p<0.01, ***p<0.001 DEC1^-/-^ mice vs the age-matched DEC1^+/+^ mice; ##p<0.01, ###p<0.001, ns p>0.05, comparisons are shown in the figure.

To confirm that DEC1 deficiency could cause the loss of DA in the midbrain, we determined the expression of TH and DAT, the two markers of DA neuron, in the midbrain of the two types (DEC1^+/+^ and DEC1^-/-^) of mice at the age of 3, 6 and 12 months by using Western blot. As shown in Figure 3, the expression of midbrain TH and DAT in DEC1^-/-^ mice at the age of 6 and 12 months significantly decreased compared with that in the age-matched DEC1^+/+^ mice (p<0.001, 0.01), whereas the change was not significant in the 3-month-old mice (p>0.05) (Figure 3A–3C). In order to observe the function of DA neuron of midbrain, we then measured the levels of striatal DA, its major metabolites dihydroxyphenylacetic acid (DOPAC) and homovanillic acid (HVA) through high performance liquid chromatography (HPLC). The striatal DA level decreased by 53.5% and 25.8% in 6- and 12-month-old DEC1^-/-^ mice compared to that in the age-matched littermate DEC1^+/+^ mice (p<0.001, 0.05) (Figure 3D), whereas the decrease of the striatal DA level was not significant in the 3-month-old DEC1^-/-^ mice compared to that in the age-matched littermate DEC1^+/+^ mice (p>0.05) (Figure 3D). Levels of DOPAC and HVA, two major metabolites of DA, also decreased significantly in 6- and 12-month-old DEC1^-/-^ mice compared with those in the age-matched littermate DEC1^+/+^ mice (p<0.001, 0.05), but the decrease of DOPAC and HVA was not significant for the 3-month-old DEC1^-/-^ mice and the age-matched WT mice (Figure 3E, 3F). Actually, the levels of striatal DA and its metabolites (DOPAC and HVA) were lower in the 12-month-old DEC1^+/+^ mice than those in the 3- and 6-month-old DEC1^+/+^ mice (p<0.001, 0.01). The changes of DA and its metabolites were consistent with those in the age-matched DEC1^-/-^ mice. While other neurontransmitters such as 5-HT, 5-HIAA and NE were almost not significant difference between the two types (DEC1^+/+^ and DEC1^-/-^) of mice at the age of 3, 6 and 12 months (Supplementary Figure 2). These results suggested that DEC1 deficient mice showed the loss of DA neurons and DA functional defects in the SNpc.

### DEC1 deficient mice show selective apoptosis in the brain

**Figure 4:**
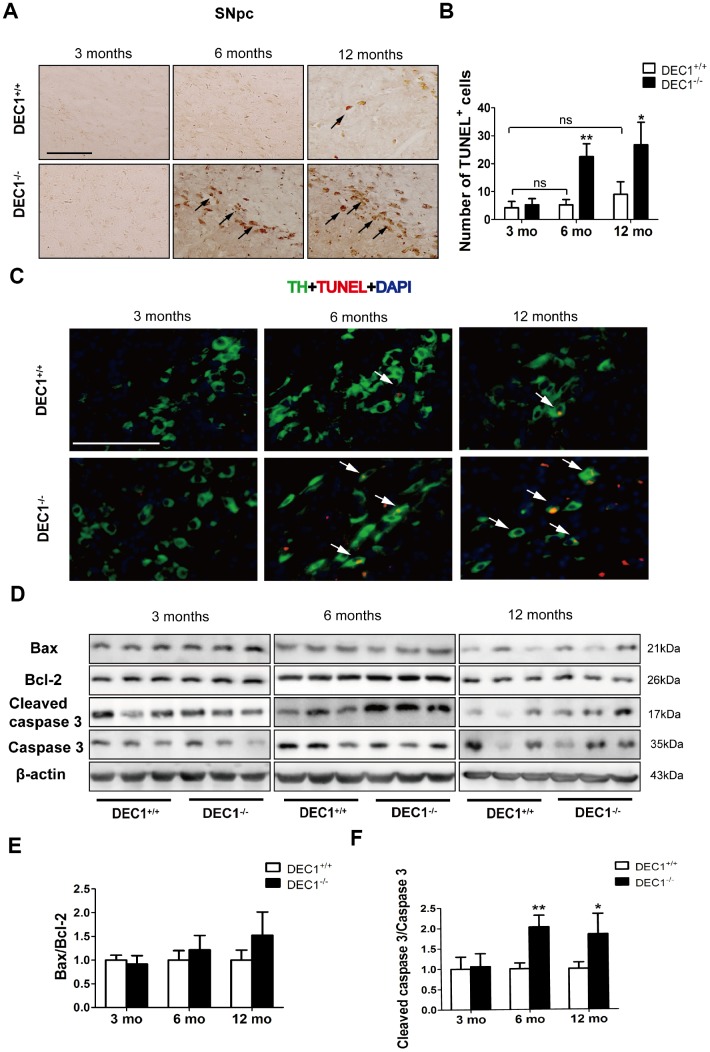
**DEC1 deficient mice show an increase in neuron apoptosis in the SNpc**. (**A**) Representative images of TUNEL^+^ cells in the SNpc of DEC1^+/+^ and DEC1^-/-^ mice at 3, 6 and 12 months(n=4 in each group). TUNEL^+^ apoptotic cells were labeled by black arrowheads. (**B**) The number of TUNEL^+^ cells (Two-way AONVA, gene: F(_1,18_)=42.961, p<0.01; age: F(_2,18_)=18.002, p<0.001; interaction: F(_2,18_)=9.031, p=0.002). (**C**) Representative images of TH (red), TUNEL (green) and DAPI(blue) in the SNpc of DEC1^+/+^ and DEC1^-/-^ mice at the age of 3, 6 and 12 months(n=4 in each group). TUNEL^+^ apoptotic cells co-expressed with TH^+^ cells were labeled by white arrowheads. (**D**) The expression of the apoptosis-related proteins at the age of 3, 6 and 12 months (n=6 in each group). (**E**) Bax/Bcl-2 (Two-way AONVA, gene: F(1,18)=4.743, p=0.069; age: F(2,18)=2.419, p=0.117; interaction: F(2,18)=2.419, p=0.117). (**F**) The cleaved caspase 3/caspase 3 (Two-way AONVA, gene: F_(1,18)_=25.945, p<0.01; age: F_(2,18)_=5.518, p=0.014; interaction: F_(2,18)_=5.518, p=0.014). The data are analyzed using t-test for the same age in two genotypes of mice and expressed as mean ± SD. *p<0.05, **p<0.01 vs the age-matched DEC1^+/+^ mice. ns p>0.05, comparisons are shown in the figure. Scale bar=100 μm.

Next, to determine whether loss of DA neurons in the SNpc was due to neuron apoptosis, we determined the number of apoptotic cells by using the TUNEL assay. As shown in Figure 4, TUNEL^+^ cells of the SNpc in the 6- and 12-month-old DEC1^-/-^ mice increased 4.3- and 3-fold, respectively, compared to those in the age-matched DEC1^+/+^ mice (p<0.01, 0.05) (Figure 4A, 4B), but the increased TUNEL^+^ cells were not significant between the 3-month-old DEC1^-/-^ mice and the age-matched DEC1^+/+^ mice (p>0.05) (Figure 4A, 4B). Meanwhile, the increased death (TUNEL^+^) cells became present in TH-positive neurons in the SNpc (Figure 4C). In addition, not any TUNEL^+^ cells in NeuN-positive ones were found in the hippocampus (Supplementary Figure 3C). These data suggested that DEC1 deficiency specifically caused the TH-positive cell death in the SNpc.

Further experiment was designed to determine the reasons of DA neuron apoptosis induced through DEC1 deficiency by evaluating the expression of apoptosis-related proteins. Notably, the cleaved caspase-3/caspase-3 in the 6- and 12- month-old DEC1^-/-^ mice increased by approximately 2 and 1.8 folds, respectively, compared to that in the age-matched DEC1^+/+^ mice (p<0.01, 0.05) (Figure 4D–4F), but the cleaved caspase-3/caspase-3 was not significantly different between the two types (DEC1^+/+^ and DEC1^-/-^) of mice at the age of 3 months (p>0.05) (Figure 4D–4F). Whereas Bax/Bcl2 was not significantly different between the two types (DEC1^+/+^, DEC1^-/-^) of mice at the age of 3, 6 and 12 months (p>0.05) (Figure 4D, 4E).

**Figure 5:**
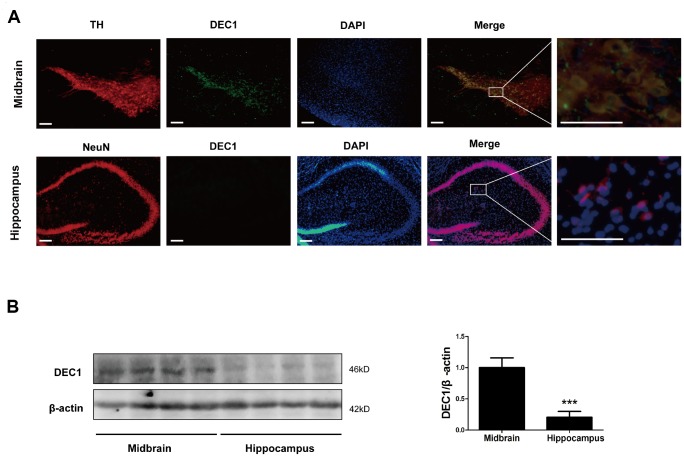
**Levels of DEC1 expression in the midbrain and hippocampus in WT mice**. (**A**) Dual staining with TH (red), DEC1 (green) and DAPI (blue) in the SNpc and hippocampus by immunofluorescence in WT mice (n=6). (**B**) DEC1 expression in the midbrain and hippocampus was analyzed by Western blotting in WT mice (n=6 in each group) at the age 6 months. The data are analyzed using t-test and expressed as mean ± SD. ***p<0.001, DEC1 expression in the hippocampus vs that in the midbrain. Scale bar=100 μm.

In order to explore the reasons of DEC1 deficiency-induced selective loss of DA neurons in the brain, we detected the expression of DEC1 in the midbrain and hippocampus of WT mice respectively. As shown in Figure 5, DEC1 was co-expressed in TH-positive cells in the SNpc and VTA (Figure 5A), and the co-expressed DEC1 and TH-positive cells took up more than 80% TH-positive cells by using immunofluorescence staining (Figure 5A). Whereas we did not find any DEC1 expression in NeuN-positive cells in the hippocampus with immunofluorescence staining (Figure 5A). Western blotting analysis showed that DEC1 expression in the hippocampus was much less than that in the midbrain (Figure 5B). The data implied that DEC1 expression in the midbrain was much more than that in the hippocampus in WT mice and DEC1 deficiency was responsible for the loss of DA in the DEC1^-/-^ mice.

### DEC1 deficiency facilitates DA neuron apoptosis of midbrain along with a decrease of PI3Kp110α, p-Ser473-Akt, p-Ser9-GSK3β and β-catenin in the 6- and 12-month-old mice

**Figure 6:**
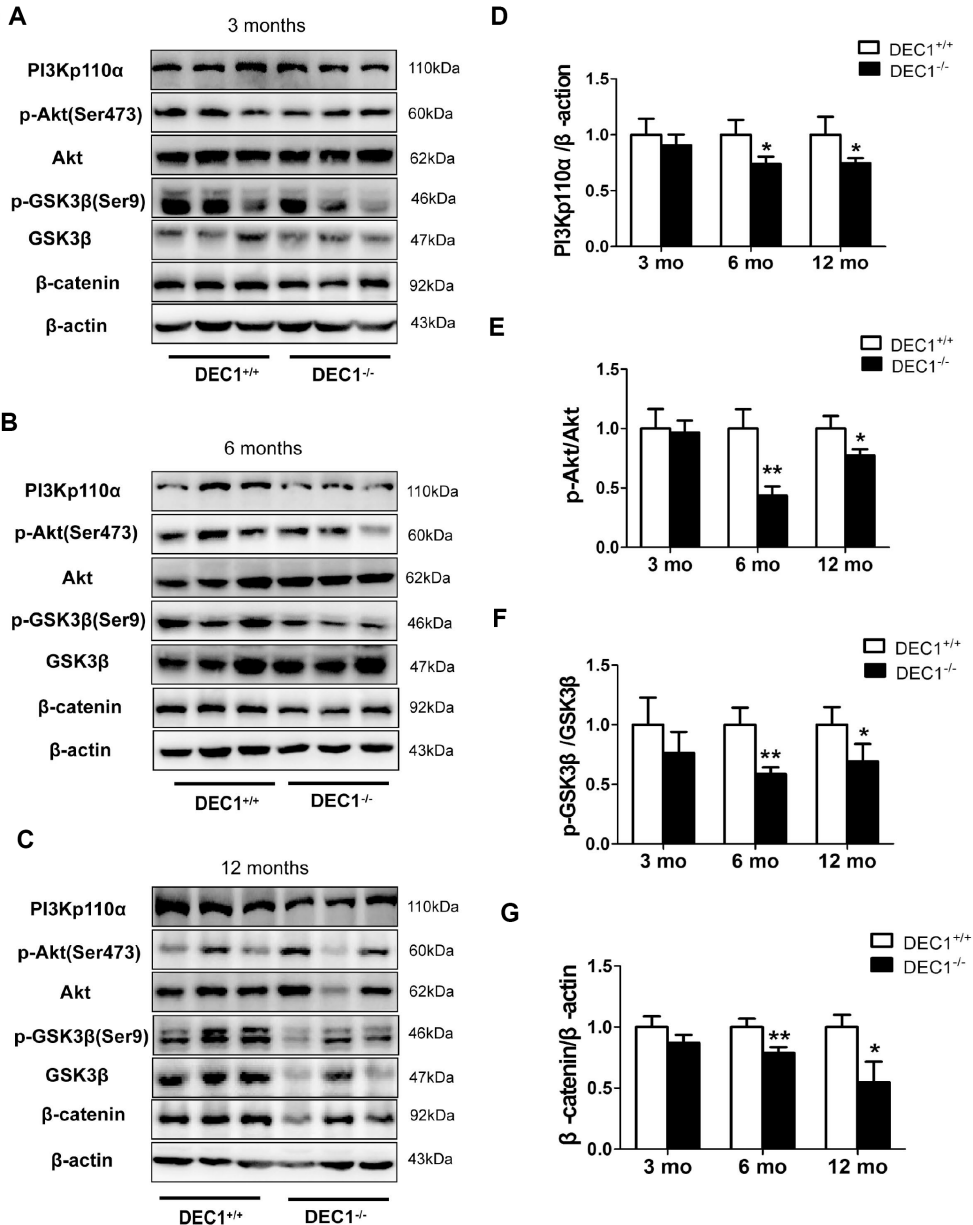
**DEC1 deficiency decreases PI3K/Akt/GSK3β pathway in the midbrain.** (**A**–**C**) Levels of PI3Kp110α and its downstream targets in the midbrain of two types (DEC1^+/+^ and DEC1^-/-^) of mice at the age of 3, 6 and 12 months (n=6 in each group) by Western blotting. (**D**) Related level of PI3Kp110α (Two-way AONVA, gene: F_(1,17)_=16.846, p=0.01; age: F_(2,17)_=1.28, p=0.303; interaction: F_(2,17)_=1.28, p=0.303). (**E**) Related level of p-Akt (Two-way AONVA, gene: F_(1,18)_=35.478, p<0.01; age: F_(2,18)_=12.597, p<0.001; interaction: F_(2,18)_=8.1, p=0.003). (**F**) Related level of p-GSK3β (Two-way AONVA, gene: F_(1,17)_=23.078, p<0.01; age: F_(2,17)_=0.635, p=0.542; interaction: F_(2,17)_=0.635, p=0.542). (**G**) Related level of β-catenin (Two-way AONVA, gene: F_(1,17)_=47.478, p<0.01; age: F_(2,17)_=6.065, p=0.01; interaction: F_(2,17)_=6.065, p=0.01). The data are analyzed using t-test for the same age in two genotypes of mice and expressed as mean ± SD. *p<0.05, **p<0.01 vs the age-matched DEC1^+/+^ mice.

To explore the mechanisms underlying DEC1 deficiency-induced apoptosis, we examined the expression of PI3Kp110α, p-Ser473-Akt, p-Ser9-GSK3β and β-catenin of midbrain in the two types (DEC1^+/+^ and DEC1^-/-^) of mice at the age of 3, 6 and 12 months. As shown in Figure 6, the levels of PI3Kp110α, p-Ser473-Akt, p-Ser9-GSK3β and β-catenin of the midbrain decreased significantly in the 6- and 12-month-old DEC1^-/-^ mice compared to those in the age-matched DEC1^+/+^ mice (p< 0.01, 0.05) (Figure 6B–6G), but not in the 3-month-old mice (p>0.05) (Figure 6A, 6D–6G). These data indicated that the inactivation of PI3K/Akt and the activation of GSK3β signaling which decreased β-catenin in the 6- and 12-month-old mice, were involved in the DA neuron apoptosis in DEC1-null mice.

### Lithium chloride rescues the DA neuron loss of midbrain in the 6-month-old DEC1 deficient mice

**Figure 7:**
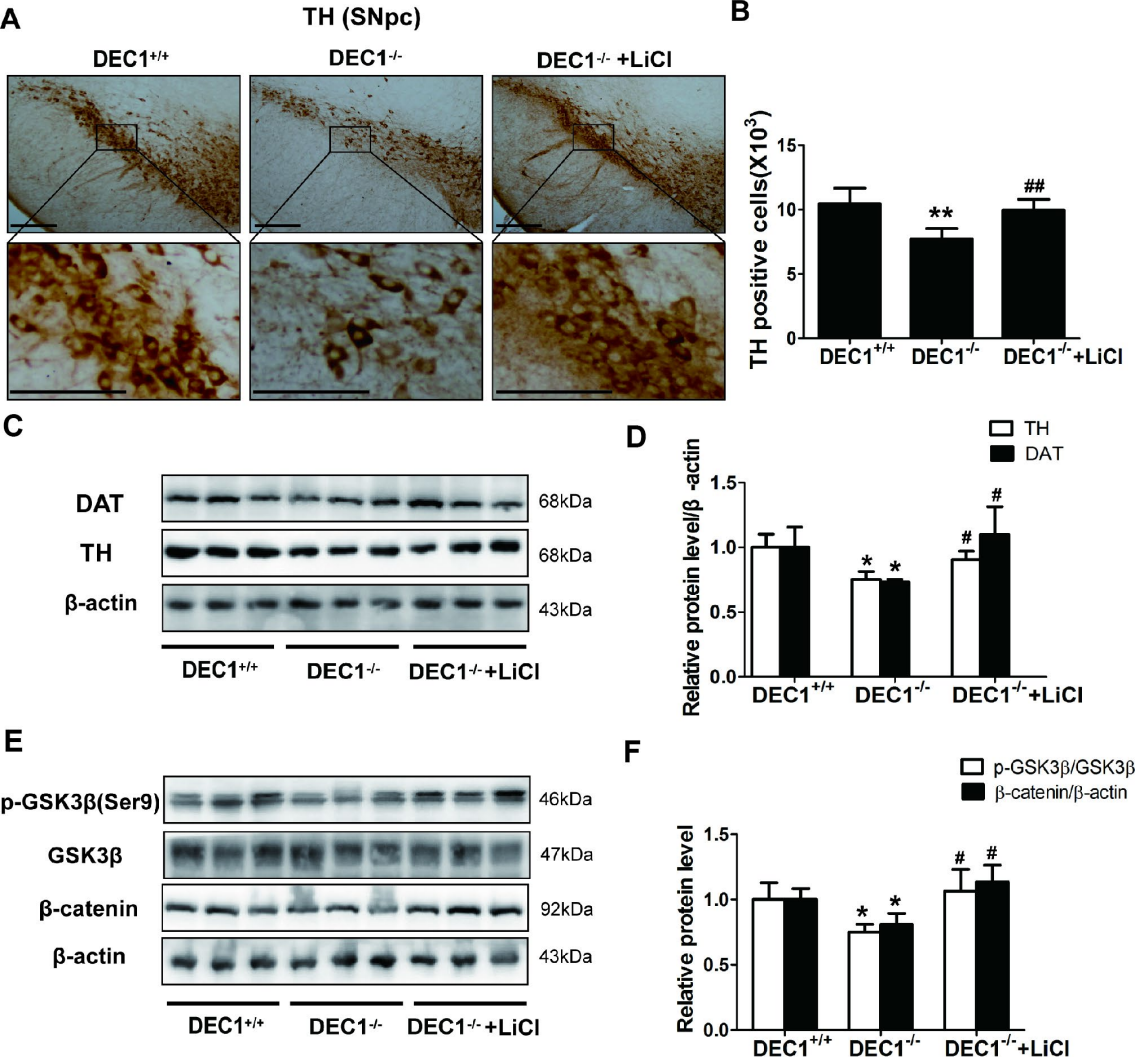
**LiCl rescues the DA neuron loss in the midbrain in the 6-month-old DEC1-null mice**. (**A**) Immunohistochemical staining of TH^+^ DA neurons in the SNpc of DEC1^+/+^, DEC1^-/-^ and DEC1^-/-^ treated with LiCl groups (n=4 in each group). (**B**) Stereological counts of TH^+^ cells in the SNpc of DEC1^+/+^, DEC1^-/-^ and DEC1^-/-^ treated with LiCl groups (Two-way AONVA, gene: F_(1,12)_=19.645, p=0.001; LiCl: F_(1,12)_=13.443, p=0.003). (**C**, **D**) TH (Two-way AONVA, gene: F_(1,7)_=17.428, p=0.004; LiCl: F_(1,9)_=7.569, p=0.028) and DAT (Two-way AONVA, gene: F_(1,9)_=8,816, p=0.016; LiCl: F_(1,9)_=16.653, p=0.003) expression in the midbrain of DEC1^+/+^, DEC1^-/-^ and DEC1^-/-^ treated with LiCl mice using Western blotting (n=4 in each group). (**E**, **F**) Levels of p-GSK3β (Two-way AONVA, gene: F_(1,7)_=7.799, p=0.027; LiCl: F_(1,7)_=10.844, p=0.013) and β-catenin expression (Two-way AONVA, gene: F_(1,9)_=10.838, p=0.009; LiCl: F_(1,9)_=31.717, p<0.001) in the midbrain of DEC1^+/+^, DEC1^-/-^ and DEC1^-/-^ treated with LiCl mice by Western blotting(n=4 in each group). The data are analyzed using t-test for the different groups and expressed as mean ± SD. *p<0.05, **p<0.01, DEC1^-/-^ mice vs the age-matched DEC1^+/+^ mice; #p<0.05, ##p<0.01, DEC1^-/-^ + LiCl mice vs DEC1^-/-^ mice. Scale bar=100 μm.

Next, we used LiCl to further investigate the mechanisms of the DA neuron loss in the midbrain induced by DEC1 deficiency**.** As shown in Figure 7, 4 weeks’ treatment with LiCl could rescue the DA neuron loss of midbrain in the 6-month-old DEC1 deficient mice (p<0.01) (Figure 7A, 7B), which was further confirmed by DAT and TH expression (p<0.05, 0.05) (Figure 7C, 7D). It should be noted that LiCl really increased the decreased p-Ser9-GSK3β and β-catenin of the midbrain induced by DEC1 deficiency in the 6-month-old mice (p<0.05) (Figure 7E, 7F), which implied that this experiment was reliable. These data supported that the decreasing PI3K/Akt/GSK3β signaling might contribute to the loss of dopaminergic neurons in DEC1-null mice.

## DISCUSSION

The loss of DA neurons in the SNpc is a major feature of the pathology of PD [21]. Although the underlying precise mechanisms of developing PD remain to be explored, increasing studies have shown that some transcriptional factors had played an important role in the neuron metabolism and maintenance of normal intracellular homeostasis [22–24]. Using different age groups of DEC1 knockout mice, we provide the evidence that DEC1 deficiency causes the loss of DA neurons, which leads to the production of PD-like phenotype in the 6-month- and 12-month-old mice, especially in the 6-month-old mice. First, we use diverse behavioral tests including OFT, BWT, RT and MWM to evaluate DEC1 deficiency in the behavior performance of the two types of mice at different age stages (3, 6 and 12 months). It is found that compared to WT mice, DEC1 deficient mice show significant motor abnormalities at the age of 6 and 12 months. The maximal difference in motor abnormalities between the two types (KO, WT) of mice becomes present in the 6-month-old mice (Figure 1A–1C). The possible reason is that both of the two types (WT and KO) of mice exhibit poor performance in motor coordination at 12-month-old (Figure 1A–1C). However, DEC1 deficiency does not significantly influence spatial learning and memory skills (Supplementary Figure 1). Actually, both of the two types (WT, KO) of mice at the age of 12 months show the greater decreased spatial learning and memory skills than those at the age of 3 and 6 months do. The latency to reach the hidden platform in WT mice at the age of 12 months increases by 1.12 folds compared with that for those at the age of 3 months. The percentage of time spent in target quadrant after removing the hidden platform and the number of crossings to pass over the platform in WT mice at the age of 12 months decrease by 59.3% and 70.6% compared with those at the age of 3 months, respectively. These results are very similar to those in some other studies, such as Carrié [25] and Frazier [26]. Second, we detect the number of TH-positive cells in the SNpc in the two types (DEC1^+/+^ and DEC1^-/-^) of mice at the age of 3, 6 and 12 months. Consistent with behavioral tests results, DEC1 deficiency decreases DA neurons (TH-positive cells) in the SNpc significantly at the age of 6 and 12 months, especially at the age of 6 months (Figure 2A, 2B). In addition, the expression of TH and DAT in the midbrain decreases significantly in the DEC1 knockout mice at the age of 6 and 12 months, especially at the age of 6 months (Figure 3A–3C), which also supports that DEC1 deficiency causes the loss of DA neurons. Similarly, DA and its metabolites (DOPAC, HVA) significantly decrease in the DEC1 knockout mice at the age of 6 and 12 months, especially at the age of 6 months (Figure 3D–3F). Why the greater difference of the loss of DA neurons between the two types of mice presents at the age of 6 months than that for those at the age of 12 months? The reason is probably that the number and function of DA neurons in the SNpc trend to descend with aging in WT (DEC1^+/+^) mice (Figure 2A left, 2B white column, Figure 3D–3F white column). These results are supported by Chen [27] and Giaime [28]. Third, using Nissl staining, we observe that the neuron number in the SNpc decreases in DEC1-KO mice at the age of 6 and 12 months compared to that in the age-matched WT mice (Figure 2C, 2D), but not in the hippocampus (Supplementary Figure 3A–3B). Moreover, the dual staining of TH/TUNEL in the SNpc and NeuN/TUNEL in the hippocampus shows that the number of TH-positive death cells increases in the SNpc in DEC1-KO mice at the age of 6 and 12 months compared with that in WT mice at the matched ages (Figure 4C). But we have not found any NeuN^+^ death cells in the hippocampus either in DEC1-KO or in WT mice at the age of 6 months (Supplementary Figure 3C). These results suggest that the loss of neurons induced by DEC1 deficiency is specific in the midbrain. It has been reported that there is expression of SHARP2 gene (DEC1 mRNA) in the midbrain and hippocampus by using hybridization in situ [18]. In this study, we find that DEC1 expression is vastly different in different brain regions. For example, DEC1 expression in the midbrain is much greater than that in the hippocampus with Western bolt (Figure 5B). We can detect DEC1 co-expressed with TH-positive cells in the midbrain but not with NeuN-positive cells in the hippocampus (Supplementary Figure 3C). The high DEC1 expression in the midbrain is the reason that the loss of neurons induced by DEC1 deficiency is specific in the midbrain.

DEC1 has critical functions in various cellular events, including cell differentiation and proliferation and inhibition of apoptosis [29, 30]. Especially, DEC1 could promote neuronal differentiation [18] and inhibit neuronal apoptosis [19]. In this study, we find that the apoptotic cells in the SNpc in DEC1 knockout mice at the age of 6 and 12 months are much more than those in the age-matched WT mice (Figure 4A–4C). Furthermore, it is found that DEC1 deficiency-induced apoptosis of DA neurons in the SNpc in DEC1 KO mice is through an increase in the caspase 3 activity but not through an increase of Bax/Bcl2 (Figure 4D–4F). These results are consistent with those in SH-SY5Y cells [19].

It is reported that aging Stra13^-/-^ mice showed massive lymphoid organ hyperplasia after 6–8 months, which is in contrast to the normal morphology and lymphocytic subpopulations in young Stra13^-/-^ mutants [14]. DEC1, as a member of clock-controlled genes (CCGs), becomes arrhythmic in the aged hypothalamic suprachiasmatic nucleus (SCN) [31]. These studies imply that the function of DEC1 might be affected by aging. Some studies have reported that aging and the degeneration of DA neurons in PD are linked by the same cellular mechanisms. For instance, some cellular markers accumulate with age, which mimics a pattern of degeneration observed in PD [32]. These markers including α-synuclein, ubiquitin as a marker of the proteasome system [33], 3-nitrotyrosine (3NT) as a marker of oxidative and glial fibrillary acidic protein (GFAP) have been implicated in dopamine neuron degeneration in PD, and have also showed age-related and region-specific changes [34]. A recent study reports T cells from PD patients recognized α-synuclein peptides, which suggests the association of PD with immune response [35]. DEC1 as a critical transcriptional mediator plays an important role in the activation of naive T cells [36]. In addition, DEC1 could promote neuronal differentiation [18] and inhibit the neuronal apoptosis [19]. These may explain DEC1 deficiency leads to the loss of DA neurons in the SNpc and elicits motor dysfunction. These notions are consistant with that alterations of locomotion activity and motor coordination are associated with DA dysfunction in animal models of neurodegeneration [37, 38]. Taken together, it is confirmed, through the loss of DA neurons, the changes of transmitters (DA, DOPAC, and HVA) and behavior tests, that DEC1 deficiency exhibits an tendency to the PD-like phenotype in mice due to the loss of DA neurons.

Our previous study has reported that DEC1 overexpression increases and DEC1 knockdown decreases the PIK3CA/Akt/GSK3β signaling in SH-SY5Y cells, inversely [19]. Here, we report that, in agreement with the loss of DA neurons, the changes of transmitters and behavior tests, the levels of PI3Kp110α, p-Ser473-Akt, p-Ser9-GSK3β, β-catenin are significantly lower in DEC1 KO mice at the age of 6 and 12 months than those in the age-matched WT mice (Figure 6). It is known that, GSK3β is an important component of the Axin complex which decreases the Wnt/β-catenin pathway [39]. In addition, PI3K/Akt can decrease the activation of GSK3β [40]. Therefore, these data support the fact that DEC1 deficiency leads to the loss of dopaminergic neurons probably involves inactivation of PI3K/Akt and activation of GSK3β signaling which causes β-catenin decrease in vivo. LiCl is an agonist of Wnt/β-catenin signaling [41] or the only clinical GSK3β inhibitor [42]. And it inhibits GSK3β activity by activating PI3K/Akt pathway [43, 44]. Next, we are eager to understand whether the Wnt/β-catenin pathway could respond to the inhibition of GSK3β activation by LiCl in the DA neurons death induced by DEC1 deficiency. As is expected, LiCl could rescue the DA neuron loss in the midbrain induced by DEC1 deficiency (Figure 7A–7D). It is noteworthy that LiCl could reverse the decreased p-Ser9-GSK3β and β-catenin caused by DEC1 deficiency (Figure 7E–7F), which protects the DA neurons from death induced by DEC1 deficiency [45]. However, LiCl treatment is not a direct evidence, as there are some inadequacies to be found out that the DA neuron loss in the midbrain induced by DEC1 deficiency is through PI3K/Akt/GSK3β signaling: (1) LiCl treatment is a systemic administration, which should probably influence other tissues and organs besides the midbrain. (2) LiCl treatment probably impacts other signaling pathways in vivo. (3) Activation of PI3K/Akt/GSK3β signaling by LiCl is mainly through increasing p-Ser9-GSK3β rather than upregulating PI3K/Akt themselves. Such notion is supported by the PI3K/Akt signaling pathway which is critical for various biological processes [46]. Similarly, the impaired PI3K/Akt signaling has been observed in neurodevelopmental and neurodegenerative diseases [47, 48]. Oppositely, the activation of PI3K/Akt signaling pathway may provide neuroprotection and anti-apoptosis [16, 19] to promote cell survival and prevent apoptosis in PD [49, 50]. However, there are some limitations in the present study. The data in the study come from the systemic DEC1 knockout mice.

In summary, we reveal the essential role of DEC1 in the maintenance of DA neuron survival. DEC1 deficient mice exhibit several key features of PD, including the loss of dopaminergic neurons in the SNpc and motor abnormalities, which is potentially involved in the PI3K/Akt/GSK3β signaling. Our findings imply that DEC1 might be a potential therapeutic target for PD.

## MATERIALS AND METHODS

### Chemical and regents

TUNEL assay kit was obtained from JIANGSU KEYGEN BIOTECH CO.LTD (Nanjing, China). ECL Western blotting detection system was from Vazyme biotech co., ltd (Nanjing, China). The following antibodies were used: anti-p-Ser473-Akt, anti-Akt, anti-PI3Kp110α (Santa, Cruz, CA, USA), anti-β-catenin (BD, San Diego, CA, USA), anti-p-Ser9-GSK3β, anti-GSK3β, anti-caspase 3, anti-cleaved caspase 3, anti-Bcl-2, anti-Bax, anti-β-actin (Bioworld, St. Louis, MN, USA), anti-TH, (Sigma-Aldrich, St. Louis, MO, USA), anti-DAT (Proteintech, Wuhan, China), anti-NeuN(Abcam, Cambridge, UK). Goat anti-rabbit IgG conjugated with horseradish peroxidase and BCA Protein Assay Kit were from Pierce (Rockford, IL, USA). DAB Peroxidase substrate kit was purchased from Vector Laboratories (Burlingame, USA).

### Animals

All the required animals in this study were bred and maintained in an SPF laboratory animal center in Nanjing Medical University. DEC1 KO mice (RBRC04841) were obtained from RIKEN BioResource Center. Heterozygous adult male mice (DEC1^+/-^) were crossed with adult female mice (DEC1^+/-^) to generate homozygous mice (DEC1^+/+^ and DEC1^-/-^) [14, 15]. Double checks (after born and before experiment) were applied to make sure the correct mouse genotype. The mouse genotyping results were presented in Supplementary Figure 4. A total ninety male mice, both of the two types (DEC1^+/+^, DEC1^-/-^) of mice at the age of 3, 6 and 12 months (n=15 in each group), were used for the behavioral tests and subsequent (such as HPLC, IHC, IF and Western blotting analyses) experiments. Eight 5-month old WT (DEC1^+/+^) male mice and sixteen age-matched DEC1 KO (DEC1^-/-^) male mice were used for LiCl rescue experiment. All mice were kept under constant environment conditions (room temperature 22 ± 2 °C, humidity of 55 ± 5 % and 12: 12 h light/dark cycle) with free water and food in the Animal Resource Center of the Faculty of Medicine, Nanjing Medical University. All the animal experiments were strictly in compliance with the experimental animal guidelines of Laboratory Animal Research Institute, and was approved by the Institutional Animal Care and Use Committee of Nanjing Medical University (IACUC:14030106).

### Behavioral examinations

Four behavioral tests were carried out under the following sequence: open-field test (OFT), beam walking test (BWT), rotarod test (RT) and morris water maze task (MWM). These behavioral tests were spaced by 24 h.

### Open-field test

Spontaneous activity of mice was recorded using a digital video camera (Winfast PVR; Leadtek Research Inc., Fremont, CA, USA) and analyzed by the video tracking software (TopScan Lite 2.0, Clever Sys, Reston, VA, USA). For each trial, mice were placed at the center of a clear, open-top, square plexigla box (40 × 40 × 35 cm^3^) and allowed to freely explore the walking route for 5 min. Total traveled distance (mm/5min) for each mouse in the box was recorded [51]. To avoid interaction between mice, the box was thoroughly cleaned with cotton pad wetted with 70% ethanol after each trial.

### Beam walking test

Mice were placed head upward on the top of a vertical wooden rough-surfaced pole (50 cm in length and 1 cm in diameter). All mice were trained for 2 consecutive days to traverse the beam. On the third day, each mouse was given five trials, and the total time for mice to turn downward and climb to the ground was measured. The animals were tested with a rest of 1 min between each trial [52].

### Rotarod test

An accelerating rotarod was used to measure the ability of forelimb and hindlimb motor coordination and balance in the mice. The mice were placed on the rotating rod training at an accelerated constant speed with a maximum (20 rpm) for 300 s in consecutive 3 days. On day 4, motor coordination was assessed on the rotarod with the maximum speed (30 rpm) for 300 s. The latency time taken for the mice to stay on the rod was recorded. The animals were tested three times with a rest of 20 min between each trial. The results were analyzed by Rota-Rod microprocessor 47600 (Ugo Basile, Biological Research Apparatus, Varese, Italy) [53].

### Morris water maze task

The water maze task was consecutively performed to evaluate animal spatial learning and memory. Mice were placed into a black-colour pool (diameter = 100 cm) filled with water (depth : 40 cm; temperature: 20 ± 1 °C). For the first 5 days’ training, a cylindrical platform (diameter = 7 cm) was placed 0.5 cm below the surface of water. Each mouse was randomly released into one of the four quadrants and allowed to swim for 60 s. Four trials were conducted each day with an interval of 30 min. The latency (s) to reach the platform were recorded for all the trials. If one mouse could not reach the platform within 60 s, the experimenter gently assisted the mouse onto the platform and allowed it to remain there for 15 s. On day 6, a probe trial was performed by removing the platform. The mouse was released from the opposite quadrant relative to the previous location of the platform and allowed to swim freely for 60 s. The percentage of swimming time spent in the target quadrant and the number of mice passing over the quondam platform were determined [54].

### Lithium chloride rescue experiment

Eight 5-month old WT (DEC1^+/+^) male mice and sixteen age-matched DEC1 KO(DEC1^-/-^) male mice were divided into three groups (DEC1^+/+^, DEC1^-/-^ and DEC1^-/-^ treated with LiCl, 8 mice in each group). Mice in the DEC1^-/-^ treated with LiCl group were injected intraperitoneally with lithium chloride (LiCl, 200mg/kg/day) for 4 weeks. While mice in DEC1^+/+^ and DEC1^-/-^ groups were injected intraperitoneally with the same volume of PBS (solvent) for 4 weeks. Twenty-four hours after the last injection, mice were killed by anesthetizing with chloral hydrate (400 mg/kg, i.p.). Brain slices or protein samples in midbrain were prepared for further experiments.

### High performance liquid chromatography (HPLC) analysis

Dissected striatal tissues of mice were prepared for the measurement of DA, dihydroxyphenylacetic acid (DOPAC), homovanillic acid (HVA), 5-hydroxytryptamine (5-HT), 5-hydroxyindoleacetic acid (5-HIAA) and norepinephrine (NE) with HPLC/ECD analysis. Brain tissue homogenate with 0.1 M HClO_4_ and 0.1 mM EDTA buffer was centrifuged at 20,000 rpm for 25 min. The supernatant was injected into an autosampler at 4 °C (UltiMate 3000, ESA) and eluted through a C18 column (2.2 μm, 120 Å, 2.1 *×* 100 mm, DIONEX) with catecholamine analysis mobile phase and was detected by ESA Coulochem III electrochemical detector. The mobile phase consisted of 90 mM NaH_2_PO_4_, 50 mM citrate, 1.7 mM 1-octanesulfonic acid, 50 μM EDTA, and 10 % acetonitrile.

### Brain slice preparation

Mice were anesthetized with chloral hydrate (400 mg/kg, i.p.) and perfused with 4% paraformaldehyde (PFA). The brains were removed and immersed in 4% PFA at 4 °C overnight and then processed for the gradient dehydration. After that, 30-μm-thick frozen brain sections (consisting of 14-15 sections) passing through the SNpc region of the brain were obtained by Leica freezing microtome.

### Immunohistochemical studies and quantitative evaluation

Brain slices were incubated with mouse anti-TH antibody (1:4000) at 4 °C overnight and followed by mouse horseradish peroxidase-conjugated secondary antibody for 1 h at room temperature. Slices were then incubated with chromogenic DAB substrates and examined for the colour change within 1-2 min. For TH cell counting, stereological analyses were performed under an Olympus DP70 microscope (200×) (Olympus America Inc., Melville, NY). The total number of immunoreactive cells in the entire extent of the SNpc was counted from 5 mouse brains per group. Each brain contained 12 serial sections at 3 intervals. The stereologer blinded to treatment groups were selected to analyze the histology for each experiment.

### Immunofluorescence

Brian sections were incubated with mouse anti-TH(1:4000) or mouse anti-NeuN(1:500) and rabbit anti-DEC1(1:500) followed by goat anti-mouse TRITC (red) (1:1000) and goat anti-rabbit FITC (green) (1:1000). Sections were washed with PBS, mounted on coverslips, and then analyzed by fluorescent microscope (Olympus, Japan) (Acquisition software: DP2-BSW).

### Western blotting

The mice were decapitated under deep anesthesia with chloral hydrate. Brain sections were quickly isolated and mouse midbrains were homogenized in a lysis buffer. The homogenate was centrifuged at 12,000 rpm for 15 min at 4 °C. The protein concentration was determined by a BCA Protein Assay Kit according to the manufacturer’s instructions. Equal amounts of protein were separated by 10 % SDS-polyacrylamide gel electrophoresis and transferred to nitrocellulose membrane by a Bio-Rad miniprotein-III wet transfer unit (Bio-Rad, Hercules, CA, USA). The membrane was blocked with 5 % non-fat milk for 2 h at room temperature. Blots were incubated with primary antibodies against TH (1:8000), β-actin (1:4000), DAT (1:2000), p-Ser473-Akt (1:1000), Akt (1:2000), PI3Kp110α (1:1000), β-catenin (1:2000), p-Ser9-GSK3β (1:1000), GSK3β (1:2000), caspase 3 (1:1000), cleaved caspase 3 (1:1000), Bcl-2 (1:1000), and Bax (1:1000) at 4 °C overnight. After being washed with TBST for three times, the membrane was incubated with appropriate horseradish peroxidase-conjugated secondary antibodies for 1 h at room temperature. The protein bands were visualized with the ECL Western blotting detection system according to the manufacturer’s instructions. The chemiluminescent signal was captured by Image Analysis software (NIH), and the relative protein level is represented as interest protein/β-actin.

### TUNEL assay

DNA fragmentation was evaluated with the TUNEL method. Brain sections containing SNpc were chosen to quantify apoptotic cells. Slices were permeabilized with 0.1% Triton X-100 for 5 min at room temperature. After being washed with PBS for 3 times, brain sections were processed with TUNEL assay kit according to the manufacturer’s instructions. TUNEL-positive cells were counted under a high power microscope (×200) (Olympus DP70, Japan) from 2 sections for each mouse, and the average number of apoptotic cells in the SNpc was gotten from 5 mice. In TH/TUNEL in the SNpc and NeuN/TUNEL in the hippocampus dual staining experiments, sections were processed with TUNEL reaction buffer at 37 °C for 1 h. After being labeled with Streptavidin-TRITC (Red) for an additional 30 min, the sections were incubated with primary antibodies (anti-TH or anti-NeuN) overnight at 4 °C. After that, the sections were incubated with appropriate secondary antibodies labeled with FITC (Green) for 1 h at room temperature. The nuclei were stained for 20 min by DAPI Staining. Dual staining (TH/TUNEL or NeuN/TUNEL) were monitored by fluorescent microscope (Olympus, Japan) (Acquisition software: DP2-BSW).

### Nissl staining

Brain sections were incubated with 0.4% cresyl violet acetate containing 0.1% glacial acetic acid for 30 min at room temperature. Slices were mounted on cover slips after being immersed in 95% ethanol for colour separation. Nissl body was observed by a conventional light microscope (Olympus DP70, Japan). We counted Nissl staining neurons of SNpc and hippocampus from two representative levels of 5 brains for each group, as described previously [55, 56]. Neurons were imaged and counted using the Olympus DP70 microscope (100× or 200×). Experimenters blind to the animal groups were selected to count the neurons of the entire structure of interest.

### Statistical analysis

The data are represented as mean ± SD. All the statistical comparisons were performed on data from at least 5 independent samples. Statistical analysis was processed by two-way ANOVA followed by Turkey’s posthoc test and paired comparisons were analyzed by t-test [28, 57] for the same age in the two genotypes of mice. The data were conducted using SPSS software, version 22.0 (SPSS Inc., USA). Difference was considered to be significant with p < 0.05.

## Supplementary Material

Supplementary Figures
