# Supplementary material for: The loss of dopaminergic neurons in DEC1 deficient mice potentially involves the decrease of PI3K/Akt/GSK3β signaling
Source: Aging (Albany NY). 2019 Dec 28;11(24):12733–53. doi: 10.18632/aging.102599 (PMC6949058; doi:10.18632/aging.102599)
Supplement: Supplementary Figures [file aging-11-102599-s001..pdf]

SUPPLEMENTARY FIGURES

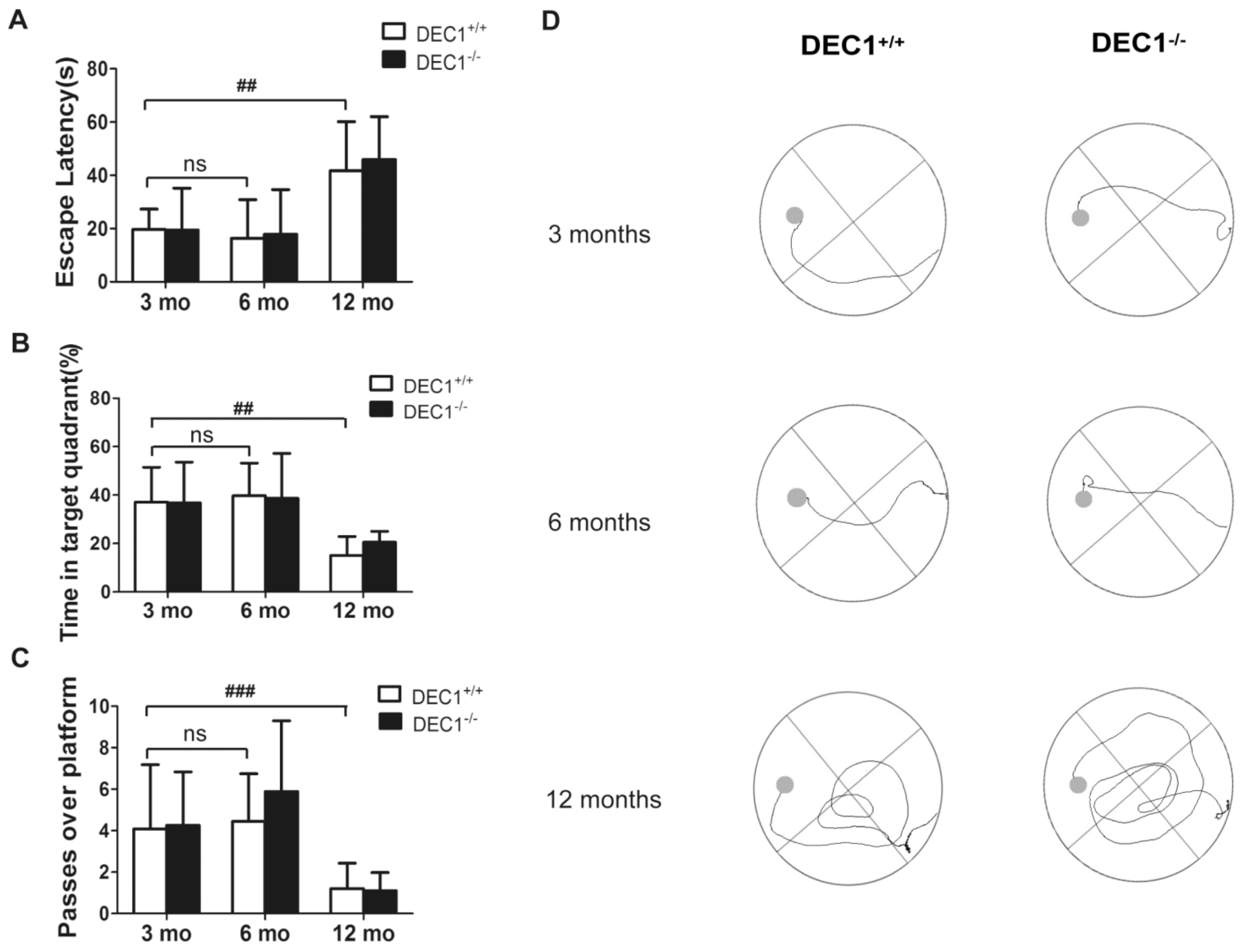

Supplementary Figure 1. DEC1 deficiency does not affect spatial learning and memory in mice. (**A**) The latency to reach the hidden platform in $DEC1^{+/+}$ and $DEC1^{-/-}$ mice at the age of 3, 6 and 12 months by using the morris water maze (MWM) test (Two-way AONVA, gene: $F_{(1,55)}$=0.277, p=0.6; age: $F_{(2,55)}$=24.701, p=<0.001; interaction: $F_{(2,55)}$=0.149, p= 0.862). (**B**) The percentage of time spent in target quadrant after removing the hidden platform (Two-way AONVA, gene: $F_{(1,55)}$=0.149, p=0.701; age: $F_{(2,55)}$=14.969, p<0.001; interaction: $F_{(2,55)}$=0.339, p= 0.741). (**C**) The number of crossings to pass over the platform(Two-way AONVA, gene: $F_{(1,54)}$=0.834, p=0.365; age: $F_{(2,54)}$=2.863, p<0.001; interaction: $F_{(2,54)}$=0.709, p=0.497). (**D**) Illustrations of representative swimming tracks of the mouse searching for the underwater platform after training five days. The dot indicates the position of the hidden platform. The data are analyzed using t-test for the same age in two genotypes of mice and expressed as mean ± SD (n=12 in each group). ##p<0.01, ###p<0.001, ns p>0.05, comparisons are shown in the figure.

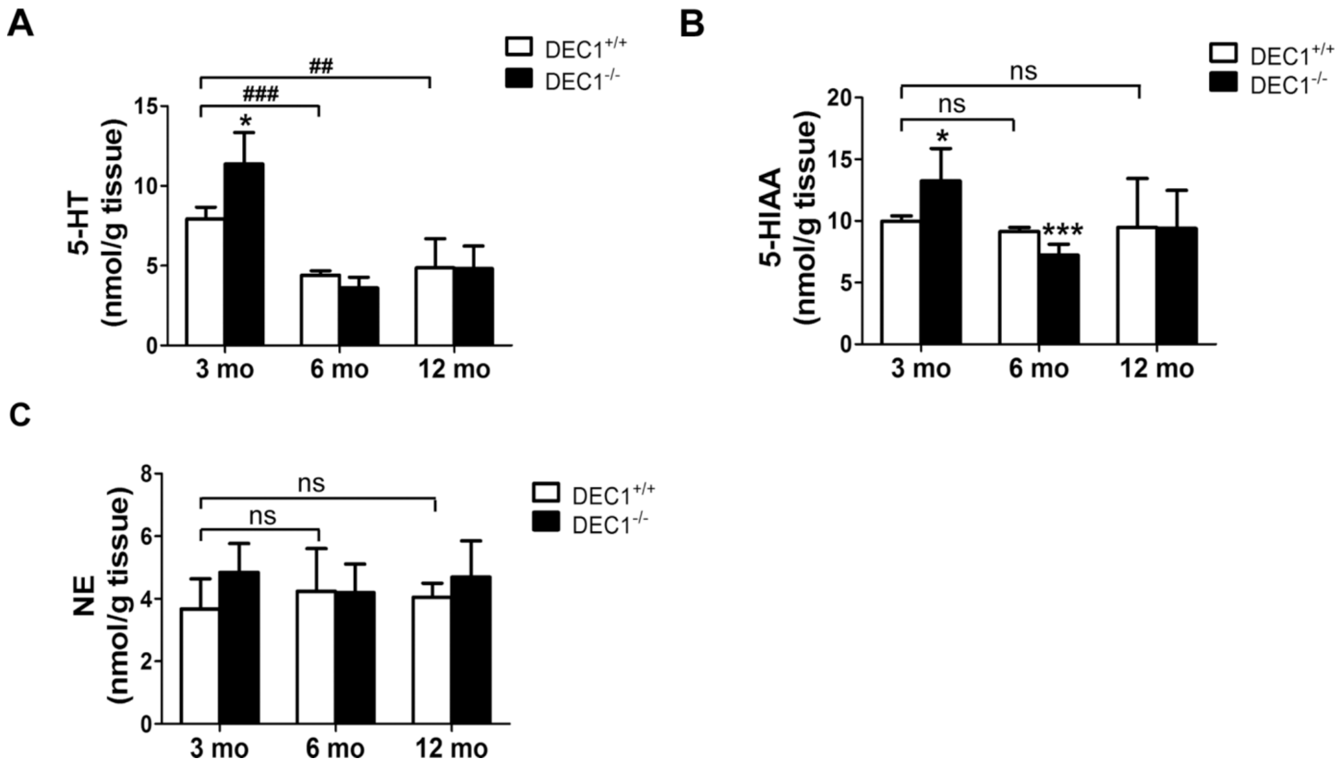

Supplementary Figure 2. Neurontransmitters (5-HT, 5-HIAA and NE) in mouse midbrain. (**A**–**C**) 5-HT, 5-HIAA and NE were analyzed by high performance liquid chromatography (n=6 in each group). (**A**) 5-HT (Two-way AONVA, gene: $F_{(1,36)}$=5.197, p=0.029; age: $F_{(2,36)}$=65.153, p<0.001; interaction: $F_{(2,36)}$=10.669, p<0.001). (**B**) 5-HIAA (Two-way AONVA, gene: $F_{(1,31)}$=0.393, p=0.535; age: $F_{(2,31)}$=7.105 p=0.003; interaction: $F_{(2,31)}$=4.27, p= 0.023). (**C**) NE (Two-way AONVA, gene: $F_{(1,33)}$=3.014, p=0.092; age: $F_{(2,33)}$=0.071, p=0.932; interaction: $F_{(2,33)}$=1.176, p= 0.321). The data are analyzed using t-test for the same age in two genotypes of mice and expressed as mean±SD. *p<0.05, ***p<0.001 vs the age-matched DEC1$^{+/+}$ mice. ##p<0.01, ###p<0.001, ns p>0.05, comparisons are shown in the figure.

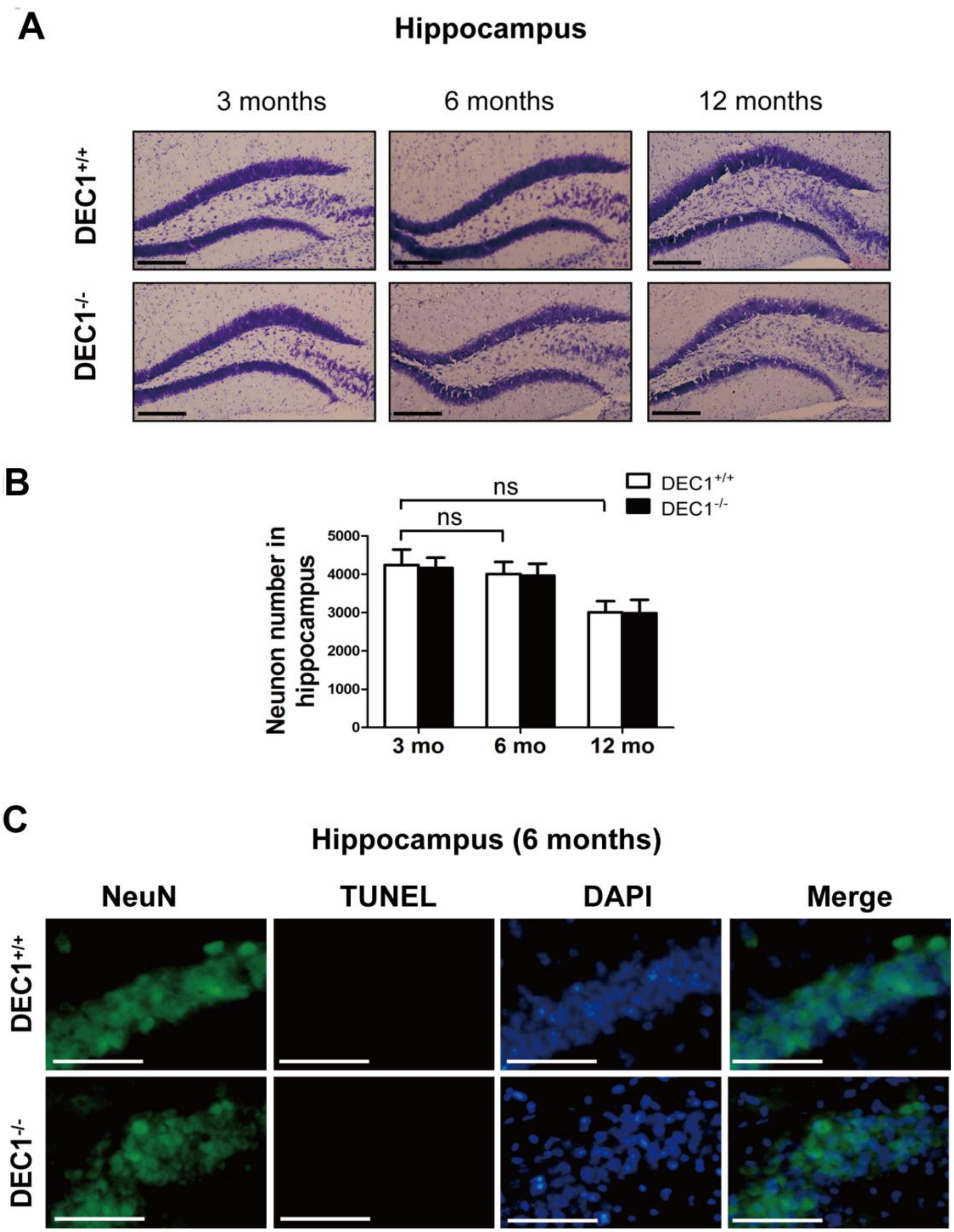

Supplementary Figure 3. Nissl staining and NeuN/TUNEL dual staining in the hippocampus. (**A**) Nissl staining in the hippocampus (n=5 in each group). (**B**) Quatification of Nissl staining in the hippocampus (n=5 in each group) (Two-way AONVA, gene: $F_{(1,22)}=0.139$, $p=0.713$; age: $F_{(2,22)}=38.612$, $p<0.001$; interaction: $F_{(2,22)}=0.018$, $p=0.982$). (**C**) Representative images of NeuN (red), TUNEL (green) and DAPI(blue) in the hippocampus of $DEC1^{+/+}$ and $DEC1^{-/-}$ mice at the age of 6 months(n=4 in each group). The data are analyzed using t-test for the same age in two genotypes of mice and expressed as mean ± SD. ns p>0.05, comparisons are shown in the figure. Scale bar=50µm.

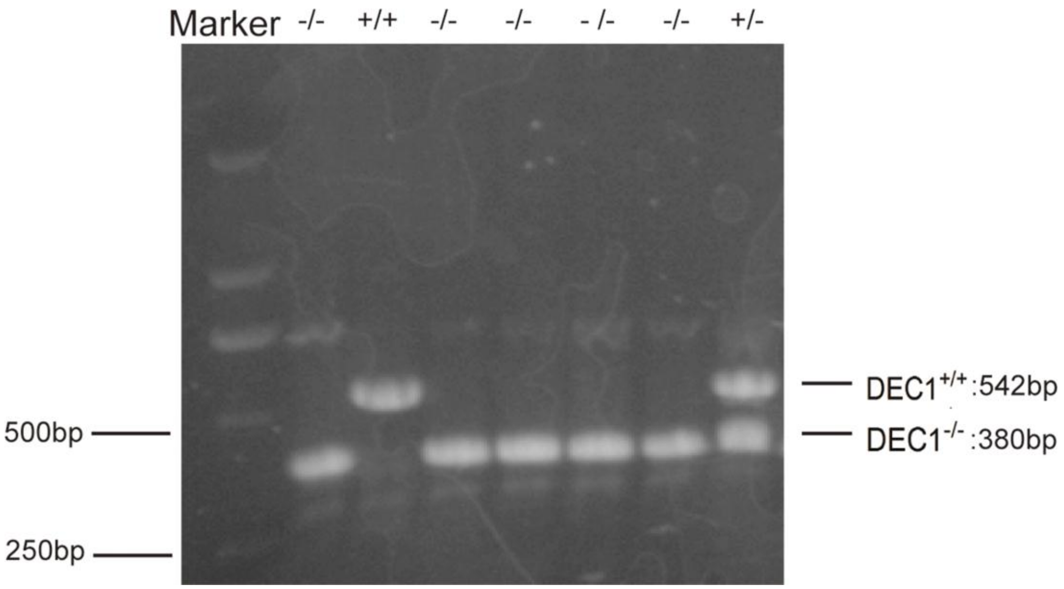

Supplementary Figure 4. Genotype identification of mice before each experiment. Tail DNA derived from $DEC1^{+/+}$ and $DEC1^{-/-}$ mice was extracted and analyzed by using PCR for genotype identification. The protocol, primers and reagents were provided by RIKEN BioResource Center.
